# Hybrid Hydrogel Composed of Hyaluronic Acid, Gelatin, and Extracellular Cartilage Matrix for Perforated TM Repair

**DOI:** 10.3389/fbioe.2021.811652

**Published:** 2021-12-24

**Authors:** Yili Wang, Feng Wen, Xueting Yao, Lulu Zeng, Jiaming Wu, Qinhong He, Huaqiong Li, Lian Fang

**Affiliations:** ^1^ ENT Department, Joint Centre of Translational Medicine, The First Affiliated Hospital of Wenzhou Medical University, Wenzhou, China; ^2^ Joint Centre of Translational Medicine, Zhejiang Engineering Research Center for Tissue Repair Materials, Wenzhou Institute, University of Chinese Academy of Sciences, Wenzhou, China; ^3^ School of Biomedical Engineering, School of Ophthalmology and Optometry and Eye Hospital, Wenzhou Medical University, Wenzhou, China; ^4^ Oujiang Laboratory (Zhejiang Lab for Regenerative Medicine, Vision and Brain Health), Wenzhou, China

**Keywords:** tissue engineering, tympanic membrane perforation, extracellular cartilage matrix, hyaluronic acid, gelatin

## Abstract

A novel series of composite hydrogels, built from the three components 1), hyaluronic acid methacryloyl (HAMA); 2), gelatin methacryloyl (GelMA), and 3), extracellular cartilage matrix (ECM), was prepared and studied regarding the possible utility in the surgical repair of damaged (perforated) tympanic membrane (TM). Noteworthy is component 3), which was harvested from the ribs of α-1,3-galactosidyltransferase-knockout (α-1,3 GalT-KO) pigs. The absence of α-1,3-galactosyl glycoprotein is hypothesized to prevent rejection due to foreign-body immunogenicity. The composite hydrogels were characterized by various aspects, using a variety of physicochemical techniques: aqueous swelling, structural degradation, behavior under compression, and morphology, e.g., *in vitro* biocompatibility was assessed by the CCK-8 and live–dead assays and through cytoskeleton staining/microscopy. Alcian blue staining and real-time PCR (RT-PCR) were performed to examine the chondrogenic induction potential of the hydrogels. Moreover, a rat TM defect model was used to evaluate the *in vivo* performance of the hydrogels in this particular application. Taken together, the results from this study are surprising and promising. Much further development work will be required to make the material ready for surgical use.

## 1 Introduction

The evidence of the role of trauma, otitis media, and iatrogenic injuries in the origin of TM perforation was increasing in recent years. Concurrently, the TM perforation therapy can be approximately segmented into two categories: surgery (autografts, allografts, and xenografts) and non-surgery (filling materials such as cotton sheet, silk, gelatin sponge) (Azimi et al., 2021; Immich et al., 2017). However, autologous graft is a conventional treatment of perforated TM. The shortage of available tissues and multiple postoperative complications limit the widespread clinical application (Seonwoo et al., 2019).

In order to overcome the limitation of tissue transplantation, various artificial materials have been tried in the field of tissue engineering. For instance, calcium alginate, silk fibroin, and chitosan extract matrix have been used for TM regeneration, but they generally have limited material properties and poor acoustic performance (Wang et al., 2021c). In recent years, 3D printing technology, melt molding technology, and other technologies have also been introduced into TM engineering, but its degradation rate, acoustic performance, anti-fouling performance of bacterial biofilm, and other aspects need more detailed clinical application research. Hence, developing an ideal artificial TM with better acoustic performance and mechanical strength, stronger adhesion, and biocompatibility is a significant challenge (Wang et al., 2021).

Recent advances in the development of biomaterials and tissue engineering have spurred the extended use of hydrogel in tissue engineering (Pan et al., 2020; Zhu et al., 2021). Compared with traditional treatments, synthetic materials afford some unique advantages including rich source and minimization of the problem of immunogenicity. In biomaterial and tissue engineering, hydrogel aroused interest for the biomimetic scaffolds, due to its structural similarity to the extracellular matrix and with high water content (Gao et al., 2021; Zheng et al., 2021). Synthetic hydrogels have good mechanical property; however, their absence of bioactivity limits their practical applications. The biological characterization of hydrogel-based biomaterials can satisfy the requirements above. Therefore, hydrogels combined to biomaterials could be an ideal scaffold material for regenerating TM.

GelMA and HAMA are often used as naturally derived biomaterials in tissue engineering and regenerative medicine, which has many advantages, such as biocompatibility, degradability, non-toxicity, large processing capacity, and photo-polymerization (Fan et al., 2020). The cellular actions of gelatin (Gel) include the promotion of cellular proliferation, differentiation, and migration, as well as adhesion, spreading, and activation (Aguilar et al., 2019). Hyaluronic acid (HA) is a ubiquitous structural component of the extracellular matrix and is abundant in the chondral and vitreous tissues. Moreover, HA has favorable hyperelasticity, strength, and biocompatibility for biomaterial applications (Xiao et al., 2019).

Animal-derived biomaterials have been a subject of growing interest in recent years. In this article, the extracellular cartilage matrix (ECM) scaffold not only has a similar biochemical composition to the natural articular cartilage ECM but also is fabricated to mimic cartilage physiological morphology with its well-oriented structure (Assuncao et al., 2020; Sekijima et al., 2014). The α-1,3 GalT-KO pigs are expected to be an excellent biomedical research model contributing to clinical medicine and reducing the widening gap between the demand and supply of human donor organs (Gasek et al., 2021; Liguori et al., 2020).

## 2 Materials and Methods

### 2.1 Preparation of HAMA

A volume of 1.00 g of sodium hyaluronate (Bloomage Biotechnology Co., Ltd., Shandong, China) was completely dissolved in 100 ml of distilled water, and then methacrylate anhydride (1.00 ml) was added to reach a final concentration of 1% (v/v) and reacted for 24 h (4°C), while 5 M sodium hydroxide was added to maintain the pH of the reaction solution between 8 and 10. After the reaction, the solution was transferred into a 12–14-kDa dialysis bag for dialysis for 3 days (4°C). Finally, the solution in the dialysis bag was frozen at −80°C for 3 h and then dried in the freeze dryer for 2 days to obtain the final product of HAMA.

### 2.2 Preparation of ECM

Fresh cartilage tissue (300 g) was collected from the ribs of a α-1, 3-Gal gene knockout pig (Yifan Dai’s lab from Nanjing Medical University, Nanjing, China), blood was removed, and then the tissue was cut into small pieces. The following steps were performed subsequently: 1) soak in 10 mM Tris–HCl solution (500 ml), stir for 24 h (45°C), and discard the supernatant; 2) soak in 0.25% (m/v) trypsin (500 ml) and stir for 24 h (37°C), then discard the supernatant (Wang et al., 2021); 3) soak in 0.1% (m/v) SDS (500 ml) for 24 h (45°C) and discard the supernatant; 4) soak in solution (PBS, 500 ml) with a protease inhibitor (Aprotinin 10 KIU/ml; Leupeptin 1 g/ml; PMSF 1 mM; 1% w/t EDTA) for 1 h (at room temperature), then discard the supernatant; 5) repeat step (3); 6) repeat step (4); and 7) after PBS washing for a few times (500 ml, 3×), freeze dry the tissue (Wang et al., 2020). After drying, the cartilage tissue was ground into powder by using the Automatic Freezing Grinding Machine and then lyophilized, and powder with particle size less than 40 μm was sieved and screened for use.

### 2.3 Preparation of Composite Hydrogel

The composite hydrogel material was prepared by mixing three types of materials, in which the HAMA content was 1% (m/v), and the contents of GelMA (Cure Gel Co., Ltd., Wenzhou, China) were 10% (m/v) and 15% (m/v), respectively, and the contents of ECM were 0% (m/v), 6% (m/v), 12% (m/v), and 24% (m/v), respectively. After the three materials were mixed evenly, 0.5% (m/v) Igracur 2959 was added as that photoinitiated and mixed well.

**FIGURE 1:**
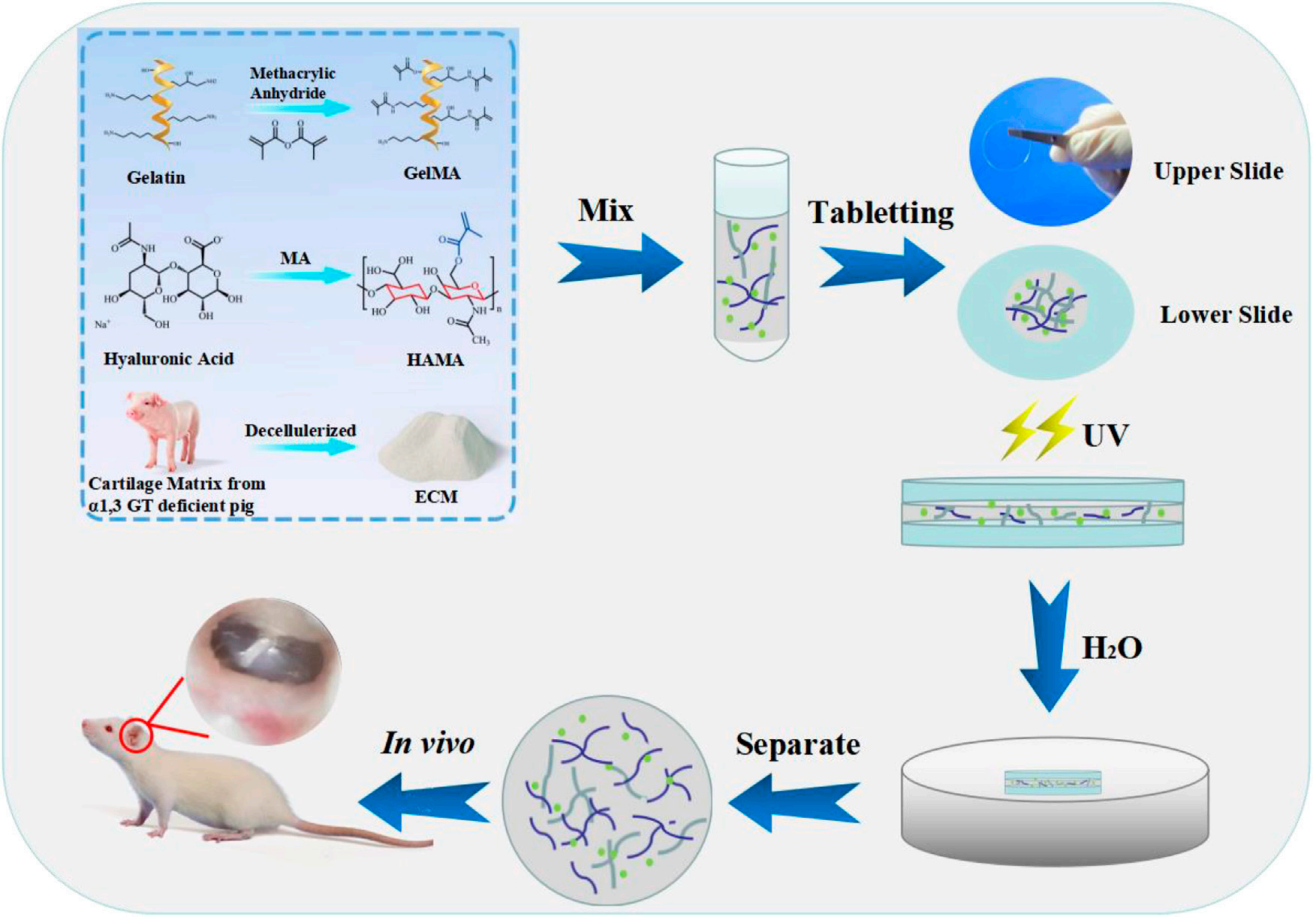
Illustration of the fabrication of the composite hydrogels. The sheet hydrogel was fabricated by photopolymerization of methacrylate gelatin, hyaluronic acid, and extracellular cartilage matrix *via* tablet.

The preparation of the polyvinyl alcohol (PVA)-coated cover plate was as follows: 30 mg PVA was dispersed in 1 ml water and stirred to dissolve completely. The cover plate was soaked in PVA solution, then taken out and placed in an oven at 60°C for drying. The prepared composite hydrogel material was dropped onto a cover plate with PVA coating (45 μl) without air bubble formation, and another cover plate with PVA coating was lightly covered immediately. The two cover plates were adhered together evenly without external force and then cross-linked under UV (365 nm, 18 mW/cm^2^) for 2 min. Subsequently, they were immersed in water until PVA was dissolved, and the cover plates fell off. The composite hydrogel scaffold (approximately 0.2 mm) was naturally separated from the cover plates (Figure 1).

The characterization analysis of the composite hydrogel includes the contents of DNA, H&E, and Masson staining; spectroscopic and internal structure are provided in the supplementary materials.

### 2.4 Characterization of Composite Hydrogel Materials

1) Swelling test

The swelling property of a hydrogel is evaluated by a weighing method. Firstly, the prepared hydrogel was weighed and recorded as M_0_. Then, the hydrogel was soaked in PBS solution (37°C, 24 h). After swelling equilibrium, the hydrogel was removed and water on the surface was wiped with a filter paper, and then mass was recorded as M_t_. The swelling rate is calculated as follows: S = (Mt−M_0_)/M_0_ × 100%, where S: swelling rate; M_t_: mass after swelling balance; and M_0_: mass before soaked in PBS (Hao et al., 2021). Experiments were done thrice per composition.

2) Degradability test

The hydrogel (5.0 mm diameter, 5.0 mm depth) was prepared by a PTFE mold. The prepared hydrogel was soaked in PBS solution to reach swelling equilibrium (24 h), weighed as W_0_, followed by soaking in 0.5 mg/ml collagenase solution (37°C), and taken out at 4, 8, 12, 24, 48, and 72 h, respectively; a filter paper was used to absorb the excess liquid on the surface and weighed as W_n_. Samples in each group were triplicate. The calculation formula of the degradation rate is as follows: D=(W_0_−W_n_)/W_0_ × 100%, where D is the degradation rate, W_0_ is the initial weight, and W_n_ is the weight at the predetermined time points.

3) Storage modulus

The hydrogel (8.0 mm diameter, 1.0 mm depth) was prepared, and the rheological mechanics of the hydrogel was accessed using a rheometer (DHR-2, USA). The constant strain was 1%, the angular frequency range was 0.1–100 rad/s, and the temperature was maintained at 37°C during the measurement. Samples in each group were triplicate.

### 2.5 Biocompatibility

#### 2.5.1 Cell Culture

Human bone marrow mesenchymal stem cells (BMSCs, Stem Cell Bank, Chinese Academy of Sciences, Beijing, China) were thawed and seeded into a 25-cm^2^ culture flask, then 5 ml of DMEM medium (containing 10% fetal bovine serum and 1% penicillin–streptomycin mixture) was added and cultured in a 37°C incubator with 5% CO_2_ (Xu et al., 2021). After 24 h, cells were examined (light microscopy); morphology and growth were assessed. When the cells covered 90% of the culture flask, the cells were passaged.

#### 2.5.2 Cytotoxicity

The hydrogel materials (50 μl) were firstly added to the 48-well plate and cross-linked by UV (samples in each group were triplicate). Then, the hydrogel materials were soaked with 200 μl of 75% ethanol for 2 h followed by PBS washing three times (30 min each) in a biological safety cabin. After PBS washing, 200 μl DMEM containing 5 × 10^3^ BMSCs was seeded into each well and cultured in an incubator (5% CO_2_, 37°C). The culture medium was changed every 3 days. Cell Counting Kit-8 (CCK-8, Beijing Solarbio Science and Technology Co., Ltd., Beijing, China) was used to detect material cytotoxicity on days 7 and 14 (Zheng et al., 2021). The absorbance value of the reactant was measured at 450-nm wavelength using a microplate reader. The cell survival rate was calculated by the following: cell survival rate =[(A_s_-A_b_)/(A_c_-A_b_)] × 100%; A_s_: absorbance of experimental well (including cells, medium, CCK-8 solution and materials); A_c_: absorbance of control well (including cells, medium, CCK-8 solution, but not materials); A_b_: absorbance of blank well (including medium, CCK-8 solution, excluding cells and materials).

#### 2.5.3 Cellular Morphology

The morphology of the cells was evaluated by live–dead and cytoskeleton staining on the 7th and 14th days after cell seeding. Acridine orange (AO) and ethidium bromide (EB) solution (1:1) were mixed in the dark according to supplier instructions. After washing with 200 μl PBS for three times, AO/EB mixture solution was added in each well and incubated for 5 min, then the solution was removed and each well was rinsed with PBS for three times (Yin et al., 2021). Cells were observed and recorded with a fluorescence microscope. For cell cytoskeleton staining, 4% paraformaldehyde was firstly used to fix the cells for 2 h followed by PBS washing, and then 200 μl of 0.1% Triton X-100 was added (10 min) to permeabilize cell membranes, and then 200 μl of Rhodamine Phalloidin was added in dark conditions for staining cell cytoskeleton (10 min). The cell cytoskeleton was stained red. After washing with PBS, DAPI was added to stain the cell nuclei for 8 min. The nuclei were stained blue. After washing with PBS, cells were observed and recorded with a fluorescence microscope.

#### 2.5.4 Cell Differentiation—Chondrogenesis-Related Gene Expression

On the 7th and 14th days post cell seeding, the total RNA of cells was extracted and reverse transcribed into cDNA as template. The relative expression levels of ACAN (forward primer: ACT​CTG​GGT​TTT​CGT​GAC​TCT, reverse primer: ACA​CTC​AGC​GAG​TTG​TCA​TGG), Sox9 (forward primer: AGC​GAA​CGC​ACA​TCA​AGA​C, reverse primer: CTG​TAG​GCG​ATC​TGT​TGG​GG), and Coll2 (forward primer: CCA​GAT​GAC​CTT​CCT​ACG​CC, reverse primer: TTC​AGG​GCA​GTG​TAC​GTG​AAC), the markers of chondrogenic differentiation, were examined by a fluorescence quantitative PCR (RT-PCR), with GAPDH (forward primer: GGC​ACA​GTC​AAG​GCT​GAG​AAT, reverse primer: ATG​GTG​GTG​AAG​ACG​CCA​GT) as a housekeeping gene (Lu et al., 2018). In addition, the ability of the material to induce stem cell chondrogenic differentiation was further verified by Alcian blue staining, as presented in the supplementary materials.

### 2.6 *In vivo* Study

The animal experiment procedure in this study was approved by the Ethics Committee of Wenzhou Institute of University of Chinese Academy of Sciences (WIUCAS 20033115, 20200331). Eight healthy rats with a body weight of 400–500 g with sensitive auricle reflex, intact TM, and clear structure were purchased for the construction of a chronic TM perforation injury model. Before operation, 10% chloral hydrate (4 μl/g) was injected intraperitoneally for anesthesia, and the external auditory canal was cleaned. After 75% ethanol disinfection, the anterior part of the TM of the bilateral TM was excised using an iris incision knife under a microscope, and the perforation area accounted for 40%–50% of the TM. The part of mucosa at the tympanic side of the perforation edge was scraped 1–2 mm using an ear micro curette, and the edge of the perforation was inverted after the four corners of the perforation were cut 1 mm. Intramuscular injection of sulfadiazine sodium (0.125 ml/kg) was used 1 day before surgery and continuously for 5 days after surgery. After the operation, the healing of the TM was observed daily after anesthesia, and the exudate was cleaned. If the perforation healed, the healed TM was opened with a microscopic ear probe, and the wound margin was inverted again. After 2 months of postoperative follow-up, the unhealed TM perforation was considered as chronic perforation, which could be used as an animal experimental model.

Composite hydrogel material 1% HAMA/15% GelMA/24% ECM was implanted into the inner TM of the TM chronic-perforated rats in our experimental group. Rats with perforated TM without implantation were set as blank control. Eight weeks post implantation, after rats were euthanatized using barbiturates, the TM repaired and the surrounding tissue were harvested immediately, observing inflammation or infection, fixed in methyl methacrylate resin after gradient ethanol dehydration, and sliced to an 8-μm-thick tissue section using a microtome for follow-up H&E staining and morphological analysis.

### 2.7 Statistical Analysis

GraphPad Prism 5 software was used for statistical analysis. Data were expressed as mean ± standard deviation, and the t test was used to compare the data of different groups. The level of statistical significance was expressed as * and *: *p* < 0.05; **: *p* < 0.01; and ***: *p* < 0.001.

## 3 Results

### 3.1 Characterization of Composite Hydrogel Materials

#### 3.1.1 Swelling

**FIGURE 2:**
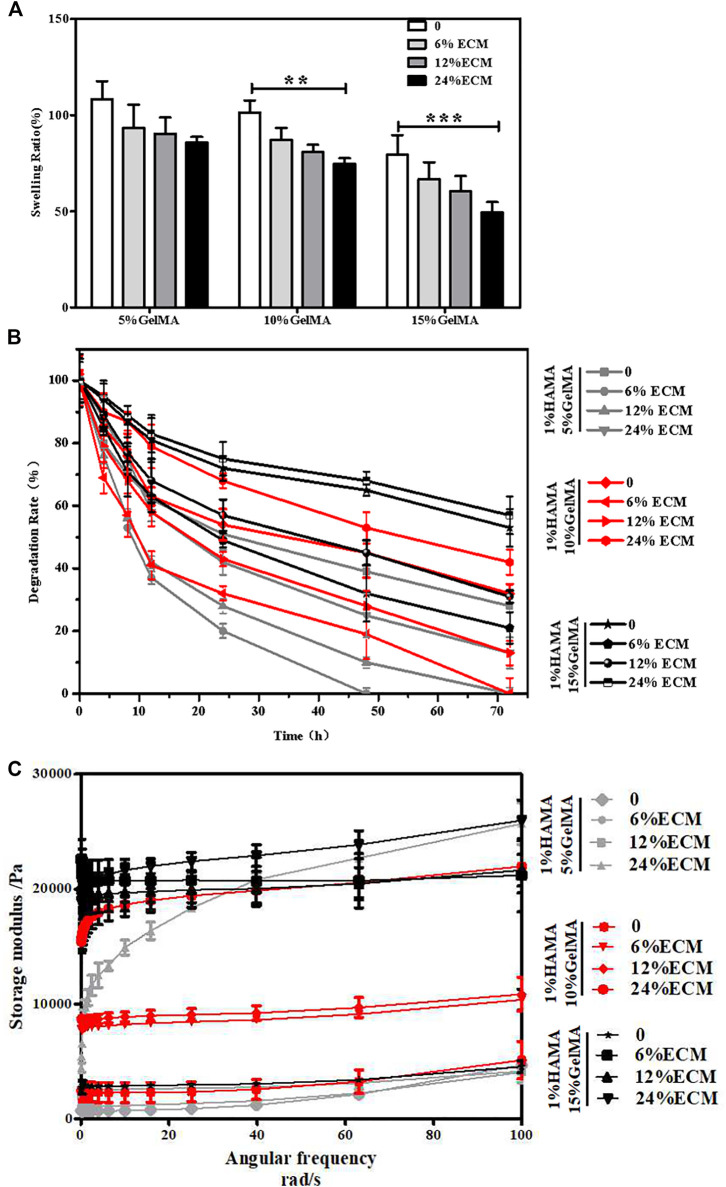
Characterization of the materials. **(A)** Swelling rate of the materials. **(B)** Degradation rate of the materials. **(C)** Storage modulus of the materials. Error bars: ± SD, *p < 0.05, **p < 0.01, ***p < 0.001.

The swelling of composite hydrogels used for TM repair in PBS solution is shown in Figure 2A. The capacity of water absorption of the material has an effect on the adhesion and growth of the cells. With the increase in GelMA and ECM contents, the water absorption capacity of the material gradually decreased, and the swelling rate reduced continuously.

#### 3.1.2 Degradability

The degradability of composite hydrogels will affect the growth of new tissues *in vivo*. If the degradation is too fast, the new tissue has not been grown, which is not conducive to the repair of damage. A too slow degradation will hinder the growth of new tissue. The degradation results showed that the degradability of the composite hydrogel was tunable (Figure 2B). With the increase in the concentration of GelMA, the degradation rate would decline, and the degradation would be further slowed down after the addition of ECM.

#### 3.1.3 Storage Modulus

The behavior of cells can be guided by the hardness and softness of hydrogels where they were cultured, such as the regulation of cell phenotypes. The results of the rheometer test showed that without decellularized ECM, the maximum storage moduli of hydrogels of 1% HAMA/5% GelMA, 1% HAMA/10% GelMA, and 1% HAMA/15% GelMA were about 4.5, 5, and 6 kPa, respectively (Figure 2C). After addition of ECM, the storage modulus of the composite hydrogel gradually increased with the increase in ECM content, and the maximum storage modulus of the 1% HAMA/15% GelMA/24% ECM group was about 24 kPa. From another point of view, these data also confirmed that the composite hydrogels have adjustable mechanical properties.

### 3.2 Biocompatibility

According to our results of physical properties of the composite hydrogel, the swelling rate of 1% HAMA/5% GelMA was high, the degradation rate was too fast, and the mechanical properties were slightly inadequate. Therefore, only 1% HAMA/10% GelMA and 1% HAMA/15% GelMA were selected in the following experiments.

**FIGURE 3:**
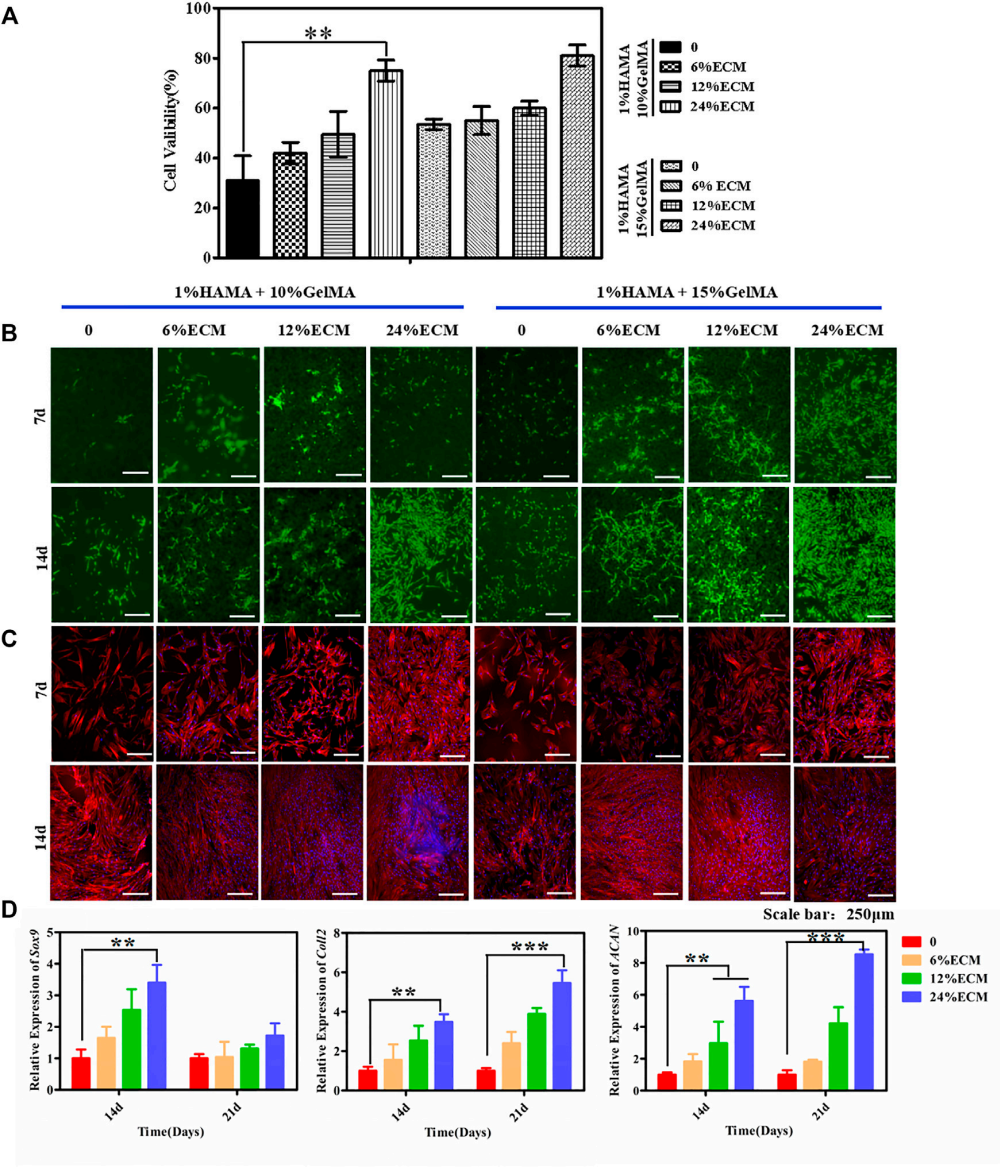
Biocompatibility of the hybrid hydrogel materials. **(A)** The CCK-8 kit was used to detect cytotoxicity. **(B)** Live/dead staining. **(C)** Cytoskeleton staining. **(D)** The chondrogenic differentiation ability of the cells was detected. The relative expression levels of Sox9, Coll2, and ACAN were detected by RT-PCR. Error bars: ± SD, *p < 0.05, **p < 0.01, ***p < 0.001. Scale bar = 250 μm.

The CCK-8 kit was used to assess the cell proliferation of the material. As shown in Figure 3A, 14 days post cell seeding on the material, the survival rate of cells showed an upward trend with the increase in GelMA and ECM contents. The reason may be that the hard matrix is in favor of cell growth, while the hardness of the hydrogel will increase with the increase in GelMA and ECM contents. In addition, some bioactive factors and components in ECM can further promote cell proliferation and adhesion. The good biocompatibility of the material was further confirmed by cell viability and cytoskeleton staining. After live–dead staining, living cells were green and dead cells were red under a fluorescence microscope. Results showed that there were many living cells on the material on the 7th and 14th days, and no dead cells were found (Figure 3B). A few dead cells may have been removed during PBS cleaning. On the seventh day, the cell proliferation on the material surface was not obvious, which may be due to the effect of glycosaminoglycan in ECM on cell adhesion. However, on the 14th day, the cells proliferated and adhered well. The cytoskeleton was stained red, the nucleus was stained blue, and the cell morphology was clearly visible by fluorescence microscopy (Figure 3C). With the further proliferation of cells, the cells interwove with each other to form a network. In terms of proliferation and spreading, cells in the ECM-containing group were better than the control.

### 3.3 Chondrogenic Differentiation

Fluorescence quantitative PCR was used to quantitatively characterize chondrogenic differentiation at the gene level. The relative expression levels of Sox9, ACAN, and Coll2, the key genes of chondrogenic differentiation, were quantitatively measured by RT-PCR on the 14th and 21st days after cell seeding, to characterize the induction of material on cell chondrogenic differentiation (Figure 3D). The expression of Sox9 was significantly upregulated at the 14th day and was most obvious in the material group with 24% ECM content. ACAN and Coll2 are the main organic components in the extracellular matrix of chondrogenic tissue and the middle and late stages of chondrogenic differentiation markers. With the increase of culture time, the secretion of ACNA and Coll2 gradually increased, and the gene expression increased significantly with the increase in the proportion of ECM content and reached the highest expression by the induction of 24% ECM. Alcian blue staining further confirmed that the material binding ECM ability had a good ability to induce stem cell differentiation *in vitro* (Supplementary Figure S4).

### 3.4 *In vivo* Study

**FIGURE 4:**
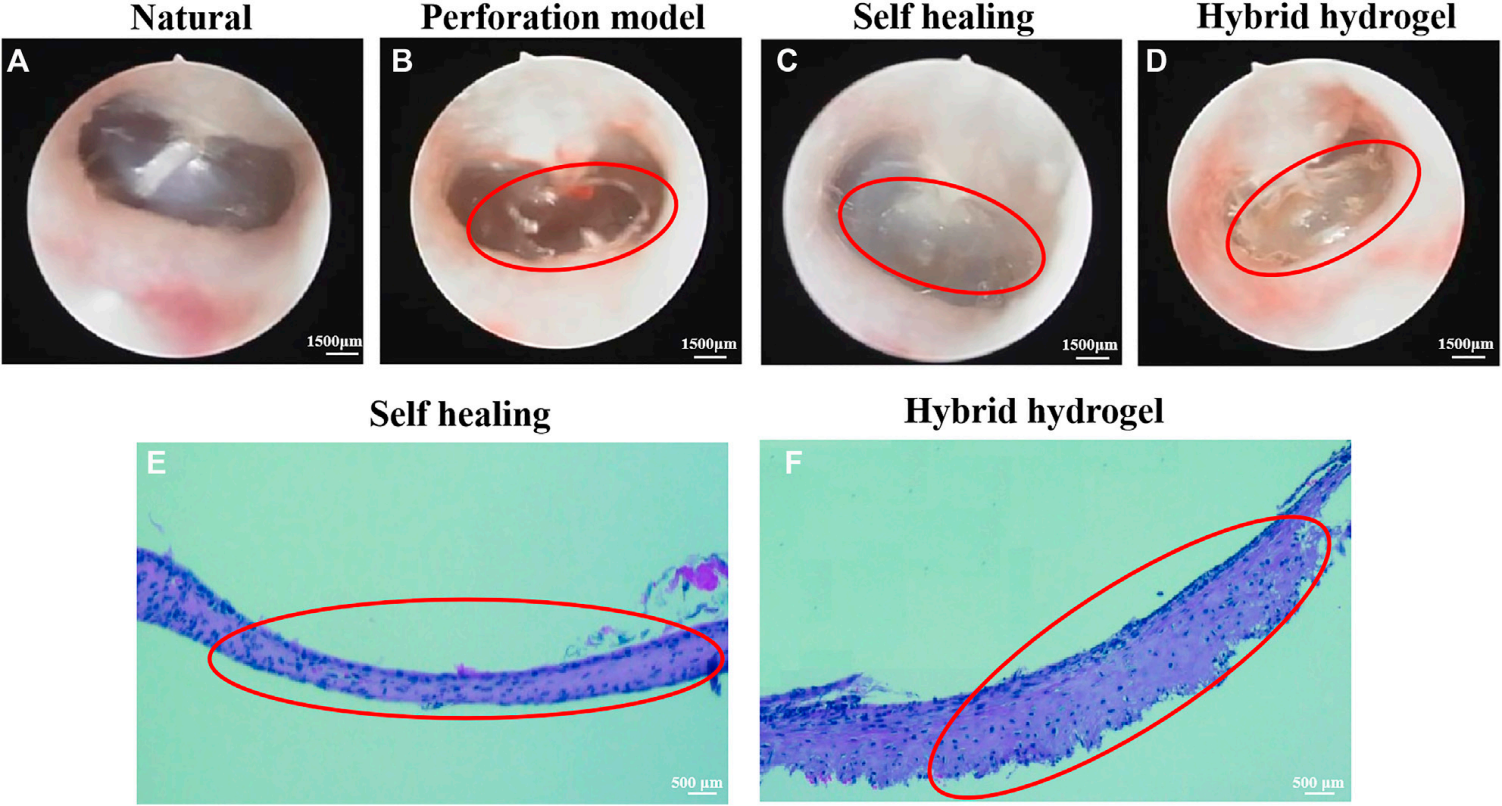
The observation images of TM structure by ear endoscopy. **(A)** Normal TM. **(B)** Perforation of TM. **(C)** Self-healing after perforation of TM. **(D)** Healing TM after filling material; H&E staining results of perforated TM. **(E)** Self-healing TM. **(F)** Healing TM after implantation of hybrid hydrogel material. The perforated TM is circled in red.

Figure 4A shows a natural transparent eardrum with a thickness of about 0.2 mm. A model of 60%–70% TM area perforated was constructed by piercing the TM tension part of rats with a microscopic ear probe (Figure 4B). The perforated site was observed weekly after surgery, and it was found that about 60% of the perforated TM injury could heal by itself within a week, but 40% of the perforated TM healing process was delayed or non-healing, and the healed TM was no more transparent under ear endoscope (Figure 4C), the tissue proliferation and adhesion in the tympanic cavity were obvious, and the healed TM was thin (Figure 4E). The TM perforation could heal within a week after implantation of the composite hydrogel scaffold (1% HAMA/15% GelMA/24% ECM) in the injured site of TM (Figure 4D). Moreover, the repair site was well recognized. There is less tissue over proliferation and adhesion in the tympanic cavity, and the TM repaired was significantly thickened, which implied a significant effect on improvement of healing (Figure 4A) (Abaci et al., 2020).

## 4 Discussion

In recent years, TM perforation has become a common disease in the department of otolaryngology; if the treatment of TM injury is not timely, it may lead to a series of diseases, which often need some medical means to assist the treatment (Gur et al., 2016). The self-healing ability of the TM exists but slowly. At present, there are various drawbacks in the treatment of TM repair (Lee et al., 2018). To overcome the problem of clinical TM perforation repair, we developed a new TM repair scaffold by combining GelMA and HAMA with a decellularized cartilage tissue matrix.

The main objective of this project is to use a new extrusion method, combining hydrogel material with acellular cartilage matrix, to create a bionic eardrum for the treatment of perforated TM. First, an innovative extrusion technique allows the material to be fabricated into a lamellar scaffold without the need for special instrumentations. In recent years, bio-printing technology has been widely used in the study of tissue engineering. For example, Kuo et al. (2018) used bio-printing GelMA to prepare a butterfly-shaped scaffold for TM defect. Although the butterfly structure is convenient to fix on the damaged site and reduces the operation of suture, it is unable to match actual defect very well. The lamellar hydrogel scaffold obtained by the innovative extrusion technology is simple and easy to operate, and the maximum storage modulus of the scaffold obtained is 24 kPa (Figure 2C), with excellent mechanical properties and certain elasticity, and therefore increase the success rate of tympanoplasty.

Hydrogels have good biocompatibility but lack biological activity and the ability to induce tissue regeneration. In this study, the incorporation of the acellular cartilage matrix mitigated this deficiency. Decellularization removed immunogenic substances from porcine cartilage, leaving mostly extracellular matrix components and some active factors, which provided a great impetus for improvement of tissue regeneration. Park et al. (Park et al., 2006) have examined the use of various hydrogels for eardrum repair, such as Carbylan (a semisynthetic glycosaminoglycan prepared by introducing an additional carboxyl group into HA), GelMA, HA, Gelfoam, Epifilm, and chondroitin sulfate. Although simple hydrogel scaffold can promote the repair of the injured site to a certain extent, the mechanical properties of the scaffold are weak, and it is easy to rupture when the pressure changes too fast or the amplitude is too large. Our composite materials enhance the mechanical properties of the scaffolds to a certain extent (Figure 2), and *in vitro* cell experiments further confirmed that ECM could promote cell adhesion and proliferation on the surface of the scaffolds (Figure 3), making composite hydrogels scaffold become a potential alternative for clinical tympanoplasty.

Compared with the self-healing group, applying a composite hydrogel scaffold in the perforated TM of rats showed that it can achieve an ideal effect, and the new tissue was well connected with the surrounding natural tissue; on the contrary, the self-healing group did not achieve complete repair and the new tissue was sticky and cloudy. Peter Luke Santa Maria et al. (Maria et al., 2016) combined heparin with a growth factor to repair TM defect. Although the repair effect was achieved after 2 months, the recovery of function still needed 1–4 months, and the whole tissue regeneration and function improvement had taken for half year, which was too long. In this regard, our scaffold was superior, and the recovery of the eardrum can be completed within 2 months without affecting the function.

## 5 Conclusion

In this study, in order to better repair the perforated TM, the widely used GelMA/HAMA hydrogel material and acellular cartilage matrix with non-immunogenicity and good biocompatibility were selected, and the mixed hydrogel material was constructed by UV cross-linking. GelMA and HAMA have been widely used in regenerative tissue engineering and clinical medicine due to their high water content and high elasticity. As a relatively new material, ECM has been decellularized to eliminate the risk of immune rejection and retain most of the extracellular matrix and collagen fibers. The addition of ECM can further induce the chondrogenic differentiation of BMSCs. The experiment of TM perforation repair further confirmed that ECM hydrogel scaffold could promote cartilage regeneration. In conclusion, our results suggest that a combination of three materials that mimic the composition of natural tissues may be a promising candidate functional material.

## Data Availability

The original contributions presented in the study are included in the article/Supplementary Material; further inquiries can be directed to the corresponding authors.
