# Supplementary material for: Hybrid Hydrogel Composed of Hyaluronic Acid, Gelatin, and Extracellular Cartilage Matrix for Perforated TM Repair
Source: Front Bioeng Biotechnol. 2021 Dec 24;9:811652. doi: 10.3389/fbioe.2021.811652 (PMC8741272; doi:10.3389/fbioe.2021.811652)
Supplement: Supplementary file 1 [file DataSheet1.doc]

**Hybrid Hydrogel Composed of Hyaluronic Acid, Gelatin and Extracellular Cartilage Matrix for Perforated Tympanic Membrane Repair**

**Yili Wang^1, 2^, Feng Wen^1, 2^, Xueting Yao^3^, Lulu Zeng^3^, Jiaming Wu^3^, Qinhong He^3^, Huaqiong Li ^1,2, 3, 4^* and Lian** **Fang ^1^, ***

^1^ Joint Centre of Translational Medicine, ENT Department, The First Affiliated Hospital of Wenzhou Medical University, Wenzhou, Zhejiang 325035, P.R. China.

^2^ Joint Centre of Translational Medicine, Zhejiang Engineering Research Center for Tissue Repair Materials, Wenzhou Institute, University of Chinese Academy of Sciences, Wenzhou, Zhejiang 325000, P.R. China.

^3^ School of Biomedical Engineering, School of Ophthalmology and Optometry and Eye Hospital, Wenzhou Medical University, Wenzhou, Zhejiang 325035, P.R. China.

^4^ Oujiang Laboratory (Zhejiang Lab for Regenerative Medicine, Vision and Brain Health), Wenzhou, Zhejiang 325000, P.R. China.

* **Correspondence:**

Huaqiong Li, [lihq@ucas.ac.cn](mailto:lihq@ucas.ac.cn); Lian Fang, [fanglian@wzhospital.cn](mailto:fanglian@wzhospital.cn).

1. **Material and Methods**

**1.1 Assessment of decellularization**

The content of DNA in cartilage before and after decellularization was determined by Quant-iT^TM^ PicoGreen® dsDNA kit. 10 mg of cartilage tissue before and after decellularization was soaked into 0.1% (m/v) TritonX-100 (1 mL) for 10 min, the supernatant was discarded. Subsequently soaked in TE buffer (200 mM Tris-HCl, 20 mM EDTA, pH 7.5) and incubated for 5 min, then discarded the supernatant. PicoGreen assay reagent was added in, and the fluorescence emission intensity was measured at 520 nm using a fluorescence microplate reader.

Whether or not the genetic material was eliminated completely was retained was assessed further by hematoxylin & eosin (H&E) staining and Masson staining. (1) The cartilage tissue before and after decellularization was soaked in 4% paraformaldehyde solution for fixation for 24 h (4 ℃); (2) The tissue was dehydrated with gradient ethanol, 1 h each time (70% → 80% → 90% → 95% → 100%); (3) The tissue was immersed in xylene solution until the tissue was nearly transparent; (4) The tissue was immersed in melted paraffin for 6 h; (5) The tissue embedded in the paraffin was cut into thin tissue slices with a thickness of 8 μm using microtome; (6) The tissue slices were stained with H&E and Masson's Trichrome according to supplier instructions; (7) observed and took images using microscope.

**1.2 Spectroscopic analysis by NMR**

Methacrylate of hyaluronic acid was characterized by ^1^H NMR (500 MHz, Bruker Avance Neo Spectrometer Durham, USA) spectrometer. D_2_O was used as a solvent, and its signal was used as a refence for the chemical shift analysis. The resonances occurred in a range of 5.2-5.7 ppm which originate from the methyl protons of the methacrylate (Mende et al.,2017).

**1.3 Internal structure**

The internal structure of the hydrogel was observed using SEM (Hitachi SU 8010, Tokyo, Japan). The prepared hydrogel was placed in liquid nitrogen for deep freezing, and then broken off from the middle with forceps, followed by lyophilization. Before SEM observation, the cross section of hydrogel was sputter-coated with Pt, using a Leica EM ACE600 instrument (Leica GmbH, Wetzlar, Germany), and the internal structure of hydrogel was observed and photographed at 20- and 200-times magnifications.

**1.4 Cell differentiation - Glycosaminoglycan (GAG) staining**

BMSCs were seeded on the hydrogel material in 48-well plates, and the content of GAG was detected by Alcian blue staining. After 7- and 14-days culture, the medium in the each well was discarded, PBS solution was added for washing 3 times, 4% paraformaldehyde was added for fixation for 24 h, then washed away with PBS. HCl solution was added to adjust the pH of solution to 1, and 1% Alcian blue was added for staining for 10 min. After washing with PBS, cells were observed and photographed under a microscope.

1. **Results**

**2.1 Decellularization**

The content of DNA in the cartilage before and after decellularization was measured by Quant-iT^TM^ PicoGreen® dsDNA kit. The results showed that the content of DNA in the cartilage before decellularization was as high as 814 ng/mg. After decellularization, the content of DNA decreased significantly, and the content of DNA in the cartilage after decellularization was only 85 ng/mg. It is a significant difference. The results of H&E and Masson Trichrome staining also confirmed the obvious effect of decellularization on DNA remove. As shown in Supplementary Figure 1, many nuclei stained blue by DAPI can be seen before the decellularization. After the decellularization, no nucleus can be seen at all, and only a lot of vacancies can be observed. According to the staining results, the extracellular matrix remained intact after the decellularization, and the tissue structure was well organized, which confirmed that the decellularization had almost no effect on the extracellular matrix structure.


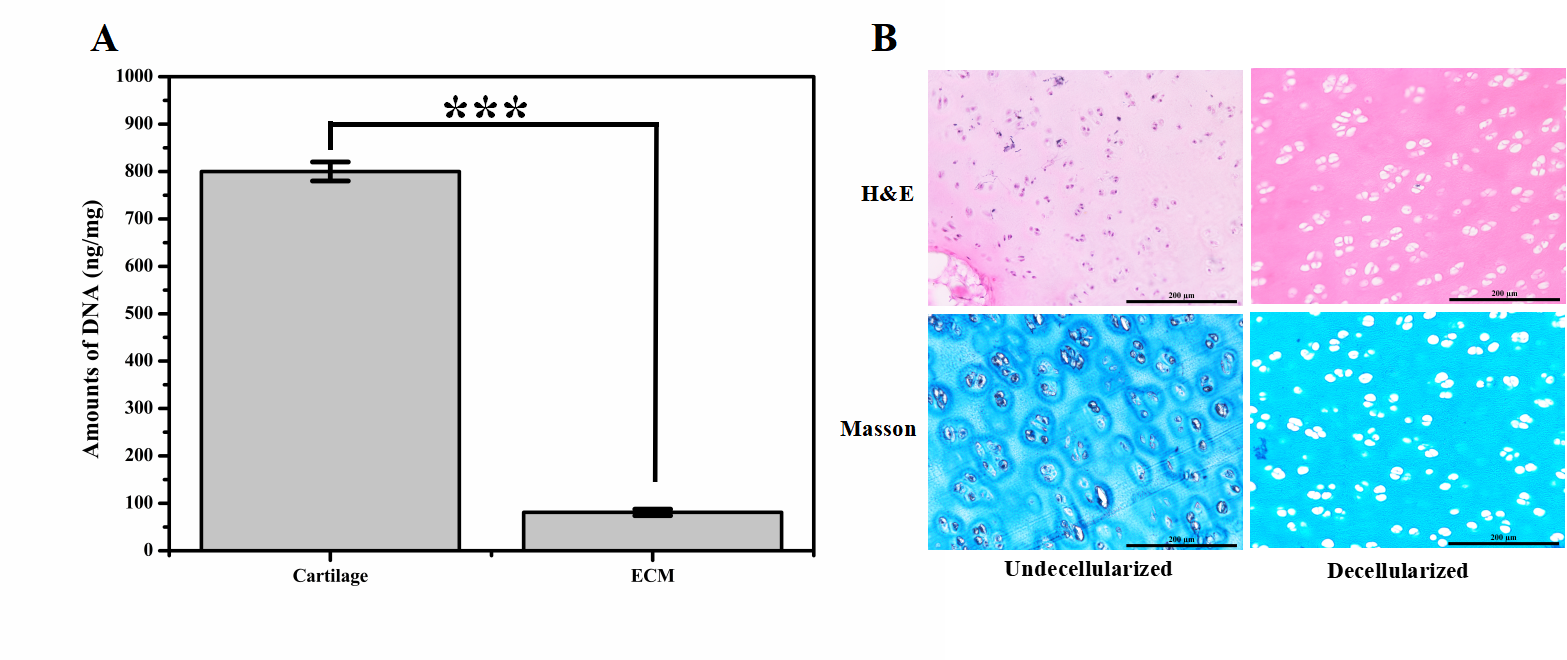


**Supplementary Figure 1.** Characterization of the materials. **A:** The result of DNA content before and after decellularization; **B:** H&E and Masson staining, the cells nuclei were completely removed, and the extracellular matrix (red) and collagen fibers (blue) were retained. Error bars: ± SD, *p < 0.05, **p < 0.01, ***p < 0.001. Scale bar = 200 μm.

**2.2 NMR analysis**

HAMA was obtained by the reaction of HA with methacrylate anhydride (MA). The degree of methacrylate acylation of HA in the reaction process had a certain influence on the mechanical properties of the reactant. Compared with the two spectra before and after the reaction with MA, the characteristic peak of C-CH3 appeared at 1.89 ppm, and the characteristic peaks of CH3=C(CH3)- appeared at 5.69 ppm and 6.12 ppm (Supplementary Figure 2). In addition, no other extra peaks were found. ^1^H NMR results proved that MA had been successfully grafted to the molecular chain of HA.


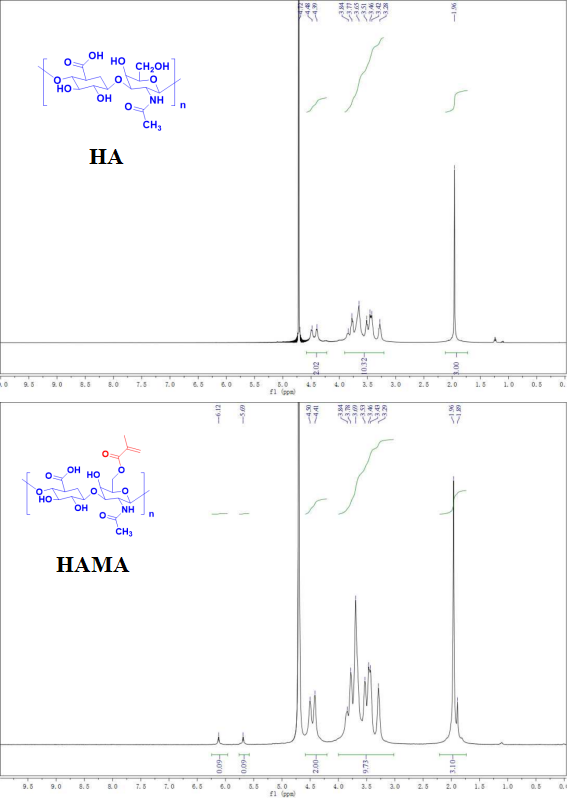
**Supplementary Figure 2.** NMR spectra of HA and HAMA. The spectra were almost identical except for two singlet signals of 5.69 and 6.12 ppm in the HAMA spectra. These peaks are related to the properties of the methacrylate groups and which will participate in the photocuring reaction.

**2.3 Internal structure**

The particle size of the decellularized cartilage powder and the internal structure of the composite hydrogel were observed by SEM, and the powder with a particle size less than 40 μm was screened for better dispersion in the hydrogel (Elomaa et al., 2020). In addition, the pore size of the composite hydrogel influences cell growth and nutrient transport. SEM results showed that the acellular cartilage powder particles were uniform, and the diameter was no more than 40 μm (Supplementary Figure 3). The internal pores of pure GelMA/HAMA hydrogel were relatively large. With the increase of decellularized cartilage content, the internal pores of the hydrogel gradually decreased, and the pores were interconnective. Decellularized ECM was evenly distributed in the hydrogel, which was conducive to the adhesion and growth of cells.


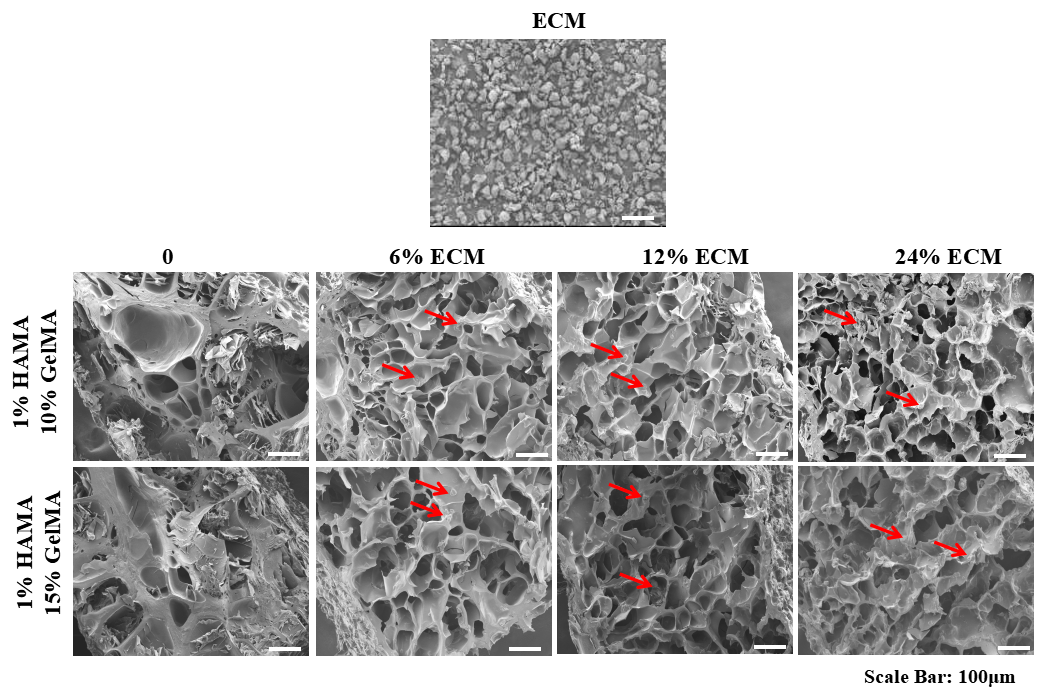


**Supplementary Figure 3.** SEM images of the materials after crosslinking. The red arrow points to where the ECM is located. Scale bar = 200 μm.

**2.4 Chondrogenic differentiation**

Alcian blue staining is a qualitative method for characterization of chondrogenic differentiation of cells, and it can specifically stain the chondrocyte extracellular matrix. Alcian blue can bind with GAG, a specific indicator of chondrogenic differentiation, to appear blue to characterize the secretion of GAG in a qualitative manner, and then to evaluate the degree of chondrogenic differentiation (Xu et al., 2019). As shown in Supplementary Figure 4, the blue stain intensity gradually deepened within 7-14 days, and the increased with the increasing proportion of ECM. On the 14st day, the area and intensity of blue stain in 24% ECM group reached the strongest, on the contrary, they were weak in 0% ECM group. In summary, the secretion of GAG increased with time, and the cumulative amount reached the highest on the 14st day. With the increase of ECM proportion, the active components in the extracellular matrix successfully induced chondrogenic differentiation, and the secretion of GAG increased significantly, showing a chondrogenic commitment.


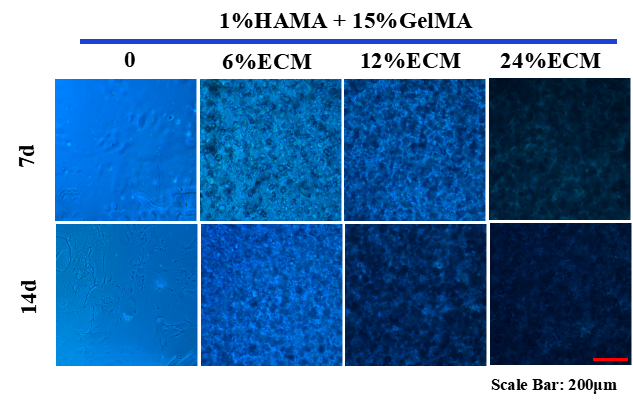


**Supplementary Figure 4.** The chondrogenic differentiation ability of the cells was detected. GAG staining was used to analyze the ability to induce chondrogenic differentiation. Scale bar = 200 μm.

**Supplementary References**

Elomaa L., Keshi E. , Sauer I., Weinhart M. (2020). Development of GelMA/PCL and dECM/PCL resins for 3D printing of acellular in vitro tissue scaffolds by stereolithography. *Mater. Sci. Eng. C Mater. Biol. Appl.* 112, 110958. doi: [10. 1016/j.msec.2020.110958](https://www.x-mol.com/paperRedirect/1249528460545515520)

Mende M., Nieger M., Brase S. (2017). Chemical Synthesis of Modified Hyaluronic Acid Disaccharides. *Chem. Eur. J.* 23, 12283-12296. doi: 10.1002/chem. 201701238

Xu S., Wu X. (2019). miR-134 inhibits chondrogenic differentiation of bone marrow mesenchymal stem cells by targetting SMAD6. *Biosci. Rep.* 39, 1-9. doi: 10.104 2/bsr20180921
